# A Year in the Bones: Investigating Bone Resorption Across a Reproductive Year in Wild Female Assamese Macaques

**DOI:** 10.1111/jpn.70108

**Published:** 2026-08-31

**Authors:** Verena Behringer, Ruth Sonnweber, Suchinda Malaivijitnond, Suthirote Meesawat, Tracy L. Kivell, Oliver Schülke, Julia Ostner

**Affiliations:** ^1^ Behavioral Ecology Department University of Goettingen Göttingen Germany; ^2^ Endocrinology Laboratory, German Primate Center Leibniz Institute for Primate Research Göttingen Germany; ^3^ Leibniz ScienceCampus Primate Cognition, German Primate Center Leibniz Institute for Primate Research Göttingen Germany; ^4^ Max Planck Institute of Animal Behavior Radolfzell/Konstanz Germany; ^5^ Department of Biology, Faculty of Science Chulalongkorn University Bangkok Thailand; ^6^ National Primate Research Center of Thailand Chulalongkorn University Saraburi Thailand; ^7^ Department of Human Origins Max Planck Institute for Evolutionary Anthropology Leipzig Germany; ^8^ Primate Social Evolution Group, German Primate Center Leibniz Institute for Primate Research Göttingen Germany

**Keywords:** CTX‐I (C‐terminal telopeptide of type I collagen), field endocrinology, maternal investment, reproductive monitoring, urinary biomarkers

## Abstract

Reproduction places considerable metabolic demands on females, particularly during pregnancy and lactation, when calcium homeostasis and bone metabolism are challenged. Urinary C‐terminal telopeptide of type I collagen (CTX‐I) offers a useful non‐invasive marker to assess bone resorption. We measured urinary CTX‐I in wild female Assamese macaques (*Macaca assamensis*) living at Phu Khieo Wildlife Sanctuary, Thailand. A total of 663 urine samples were collected from 19 adult females between June 2017 and January 2019 and analysed using a CTX‐I ELISA. Generalised additive modelling revealed a non‐linear relationship between days relative to conception and urinary CTX‐I levels, with higher values before conception and after parturition, and lower levels during pregnancy. However, this pattern remained when lactating females from the previous year (6 out of 19) were excluded, and further analyses showed that the seasonal, rather than the reproductive term, best‐explained variation in urinary CTX‐I levels. CTX‐I levels showed two annual peaks, with the main one occurring in the late rainy season (August–September). Seasonal changes in urine concentration did not account for this pattern. These findings suggest that, in the study population of Assamese macaques, bone resorption varies with season, possibly in response to environmental or endocrine cycles, rather than being primarily driven by the metabolic demands of reproduction.

## 1 Introduction

Female mammals experience substantial energy demands and physiological adjustments during reproduction (Beehner and Lu 2013; Jasienska 2020). If external resource availability is limited, energy has to be reallocated depending on the balance between competing physiological needs, with reproduction representing one of the most evolutionarily relevant processes (Thompson 2013; Jasienska 2020). From a hormonal and overall physiological perspective, mammalian and thus primate reproduction follows a continuous sequence of events: mating, gestation, parturition, lactation, post‐lactational maternal care, and maternal recovery (Gittleman and Thompson 1988). Pregnancy and lactation are energetically demanding periods in mammals, accompanied by an increase in basal metabolic rate. These costs may be buffered through energy‐saving physiological mechanisms as well as increased food intake, and/or a reduction in physical activity (Boinski 1988; Dunbar et al. 2002; Jasienska 2020; McCabe and Fedigan 2007; Murray et al. 2009; Tirado Herrera and Heymann 2004; Vasey 2005). These metabolic adjustments not only affect energy allocation, acquisition and conservation, but also pose specific challenges to bone metabolism.

The skeleton is one of the less visible, but biologically important, systems affected by reproduction. Bone is a dynamic tissue that undergoes modelling and continuous remodelling throughout life, processes essential for adapting to changing biomechanical forces, repairing microdamage, and maintaining structural integrity (Currey 2002; Kini and Nandeesh 2012; Nandiraju and Ahmed 2019). Bone is composed of cells involved in the processes of maintenance and remodelling (e.g., osteoblasts, osteoclasts, and osteocytes), embedded in an organic matrix primarily made of collagen (Boskey and Robey 2013; Kini and Nandeesh 2012). The balance between bone resorption and formation is influenced by genetic, mechanical, vascular, nutritional, and hormonal factors, all of which can change with reproductive state (Mallorie and Shine 2022; Nandiraju and Ahmed 2019; Singh et al. 2023).

In addition to their structural and metabolic functions, bones serve as a key reservoir for essential minerals, most notably calcium and phosphorus, which can be mobilised to maintain systemic mineral homeostasis. Approximately 99% of the body's calcium is stored in the skeleton, highlighting the pivotal role of bone in calcium regulation (Kini and Nandeesh 2012).

During energetically demanding life‐history phases such as pregnancy and lactation, maternal calcium requirements increase substantially to support fetal skeletal development and milk production, with lactation generally representing the more calcium‐demanding phase (Kovacs 2016). As a result, these reproductive stages are characterised by physiological adaptations in calcium metabolism to meet the demands of the fetus, infant, and mother. In pregnancy, increased calcium demand is met primarily by enhanced maternal intestinal absorption from food, with only a modest skeletal contribution (Kovacs 2016; Tsourdi and Anastasilakis 2021). In contrast, during lactation, calcium is predominantly mobilised from maternal bone through osteoclast‐mediated bone resorption and osteocytic osteolysis (Kovacs 2016; Tsourdi and Anastasilakis 2021).

Bone resorption can be quantified using biochemical markers that reflect the degradation of bone matrix components. One of the most widely used markers in both clinical and research contexts is the C‐terminal telopeptide of type I collagen (CTX‐I), a fragment generated during the breakdown of type I collagen, which accounts for over 90% of the organic bone matrix (Herrmann and Seibel 2008; Rosen et al. 2000; Vasikaran et al. 2011). CTX‐I is released into circulation in proportion to osteoclast‐mediated collagen degradation and, thus, serves as a reliable indicator of bone resorption activity (Herrmann and Seibel 2008; Urlacher et al. 2022). Due to its small molecular size, CTX‐I is metabolised and excreted via the kidneys, allowing its detection in both blood and urine (Herrmann and Seibel 2008; Komi et al. 2004). Urinary CTX‐I levels are positively correlated with serum levels (Szulc et al. 2017), and the excretion rate of these collagen fragments provides a non‐invasive marker of bone collagen degradation (Mora et al. 1998). Therefore, CTX‐I is particularly valuable in settings where blood sampling is logistically and/or ethically constrained. In non‐invasive eco‐physiology, urinary CTX‐I offers a promising tool for assessing bone resorption, especially during life‐history stages such as pregnancy and lactation, when calcium homeostasis is challenged (Behringer et al. 2025; Urlacher et al. 2022).

In humans, longitudinal studies have consistently reported a pronounced increase in maternal urinary CTX‐I levels as pregnancy progresses, with concentrations at term reaching two‐ to threefold higher levels than those observed in early gestation or prior to conception (Curtis et al. 2021; Hellmeyer et al. 2006; Kaji et al. 2007; Zhang et al. 2023). Similar patterns have been documented in several domesticated mammals, including dairy cows, pigs, goats, and sheep, in which serum CTX‐I levels increased around parturition (Liesegang et al. 2000, 2006, 2007; Van Riet et al. 2016). Extending this approach to non‐human primates, a recent study on bonobos (*Pan paniscus*) found increasing urinary CTX‐I levels towards the end of gestation in both zoo‐housed and in primiparous wild females, whereas levels in multiparous females remained comparatively stable (Behringer et al. 2025). Overall, these findings suggest a conserved physiological response of increased bone resorption during pregnancy (i.e., increased calcium demand) across a broad range of mammalian taxa.

Similar to pregnancy, lactation is associated with pronounced changes in maternal bone metabolism across mammals. In humans, urinary and serum CTX‐I levels continue to fluctuate postpartum, with levels remaining elevated compared to pre‐pregnancy or early pregnancy levels (Augustine et al. 2023; Yamaga et al. 1997). Several studies even report higher urinary and serum CTX‐I levels during lactation than in the third trimester, highlighting the substantial skeletal calcium mobilisation required to support milk production (Carneiro et al. 2010; Prentice et al. 1998). Comparable patterns have been documented in a range of domesticated mammals including sows, goats, sheep, and cows, where CTX‐I levels increase during lactation relative to pregnancy and pre‐pregnancy states (Liesegang et al. 2000, 2006, 2007; Van Riet et al. 2016). In non‐human primates, CTX‐I measurements have so far focused mainly on age‐related variation in blood samples (Li et al. 2022; Sattgast et al. 2021) and on evaluating treatment‐induced changes in urinary CTX‐I (Lindström et al. 2018). To date, however, studies in captive non‐human primates have not specifically assessed urinary CTX‐I levels in relation to lactation. Nevertheless, increases in bone turnover during lactation have been shown through alternative markers, including pyridinoline and deoxypyridinoline (Lees et al. 1998), enhanced bone remodelling activity (Hiyaoka et al. 1996), and even direct evidence of skeletal calcium depletion (Ott et al. 1999). However, in contrast to this broadly conserved pattern, urinary CTX‐I levels in wild female bonobos were higher during pregnancy than during lactation, with early lactation exhibiting the lowest CTX‐I levels across all reproductive states (Behringer et al. 2025). This pattern deviates from the expectation that lactation universally increases bone resorption due to heightened calcium demands. Instead, it suggests a distinct reproductive modulation of bone metabolism in wild bonobos, highlighting the need to account for ecological and species‐specific variation in reproduction when interpreting maternal skeletal dynamics across mammals.

Seasonal reproduction is a widespread adaptive strategy among primates, allowing females to align the energetically demanding phases of reproduction with periods of high ecological resource availability (Brockman and van Schaik 2005). One strategy is to time births so that the energetically costly lactation period coincides with or precedes seasonal peaks in food abundance, which is often referred to as income breeding (Clutton‐Brock et al. 1989; Gittleman and Thompson 1988; Stearns 1992). This strategy enhances maternal energy balance and infant survival by ensuring that critical stages of offspring development occur when nutritional resources are most abundant.

Female Assamese macaques (*Macaca assamensis*) can complete reproductive cycle—pregnancy, lactation, and weaning—within approximately 1 year (Fürtbauer et al. 2010; Touitou et al. 2021b), although interbirth intervals are typically bimodal with both ~1‐ and ~2‐year intervals observed (Shivani et al. 2025). In the well‐studied wild population at Phu Khieo Wildlife Sanctuary (PKWS) in Thailand, conceptions are tightly clustered within the mating season, and approximately 80% of births occur within a 3‐month window from April to June, following an average gestation of 165 days (Fürtbauer et al. 2010; Touitou et al. 2021a). Ovarian cycling ceases outside the breeding season, and conceptions typically occur during a female's first or second ovulatory cycle (Fürtbauer et al. 2010). Although most interbirth intervals are approximately 2 years long (2/3 of cases; Shivani et al. 2025), a substantial proportion (1/3) of interbirth intervals are close to 1 year. In the latter case females proceed through pregnancy and lactation within a single year, facilitating the potential for pregnancy again the following year. Such rapid succession of reproductive stages within a single year likely limits the opportunity for skeletal recovery between reproductive events, potentially intensifying cumulative effects on bone metabolism.

Furthermore, the habitat of the study population is characterised by seasonality in terms of pronounced variation in ambient temperature and precipitation as well as in food availability. Cold and dry weather from November to mid‐March with minimum temperatures occasionally approaching freezing in January, is followed by a long, warm to hot rainy season with an average annual precipitation of 1374 mm (Richter et al. 2016). How and to what degree food abundance for the frugivorous Assamese macaques increases through the rainy season is highly variable between years (Anzà et al. 2025; Berghänel et al. 2016; Heesen et al. 2013). Given their well‐documented seasonality and rapid reproductive turnover, Assamese macaques at PKWS offer an opportunity to assess whether bone resorption patterns in wild primates living in natural environments align more closely with domesticated species or with free‐ranging apes.

Based on prior findings in humans and domesticated mammals, we predicted that urinary CTX‐I levels would increase progressively during gestation and reach a maximum during early lactation. Alternatively, if the pattern observed in wild bonobos—characterised by lower CTX‐I levels in early lactation compared to pregnancy—is representative of free‐ranging non‐human primates exposed to natural environmental constraints, we predicted a decrease in CTX‐I levels following parturition, followed by a gradual increase as lactation progressed.

## 2 Materials and Methods

### 2.1 Study Population and Sample Collection

Demographic data and urine samples were collected as part of the long‐term study on Assamese macaques in their natural habitat at PKWS, part of a 6500 km^2^ protected forest area in northeastern Thailand, approximately between 16°5'− 16°35'N and 101°20'−101°55'E. The study site, Huai Mai Sot Yai (16°27'N, 101°38'E), ranges in elevation from 600 to 800 metres above sea level and is dominated by hill evergreen forest with scattered patches of bamboo and dry dipterocarp forests (Borries et al. 2002; Koenig et al. 2004; Kumsuk et al. 1999). We collected 663 urine samples from 19 adult females between June 2017 and January 2019 (sample distribution per female in Table 1).

**Table 1:** Distribution of urine sample collection across 19 wild female Assamese macaques, all of which gave birth in 2018, displayed per reproductive stage (pre‐, early‐, late‐ and post‐gestation). Additionally, the table indicates which females gave birth in 2017.

Female	Pre	Early	Late	Post	Sum	Infant in 2017
1	11	2	6	13	32	Yes
2	12	4	5	10	31	Yes
3	7	3	4	18	32	No
4	8	11	5	18	42	No
5	5	10	6	14	35	No
6	6	8	6	11	31	No
7	11	1	3	12	27	No
8	11	5	6	14	36	No
9	6	8	7	25	46	No
10	6	6	3	8	23	Yes
11	11	2	7	18	38	No
12	13	4	2	18	37	Yes
13	11	11	3	20	45	No
14	5	6	3	8	22	No
15	7	7	7	15	36	No
16	14	6	7	14	41	No
17	7	4	6	17	34	No
18	16	5	5	12	38	Yes
19	9	8	6	14	37	Yes
**Sum**	**176**	**111**	**97**	**279**	**663**	

Urine samples were collected opportunistically throughout the day using disposable mini‐pipettes. Only urine samples free from fecal contamination were collected. The samples were either pipetted from clean, disposable plastic bags placed underneath the females during urination or directly from vegetation. The urine was then transferred into 2 mL Eppendorf vials, which were labelled with the date, time of collection, and female ID (Touitou et al. 2021b). Specific gravity (SG) was measured immediately after collection using a handheld refractometer (Atago PAL–10S), with a few drops of urine placed on the lens. The lens was cleaned with Kimtech wipes between measurements to prevent contamination. To maintain sample integrity, the urine was kept cool in the forest using Thermos flasks filled with ice cubes. At the end of each day, the samples were stored in a freezer at the camp (−12°C) before being transported to a second freezer (−19°C) in a nearby village (Touitou et al. 2021b). The samples were kept frozen until being shipped on dry ice to the endocrinology laboratory of the German Primate Centre, Göttingen, Germany, for analysis.

### 2.2 Ethics Statement

This study was conducted in accordance with the ASAB/ABS Guidelines for the Use of Animals in Research (https://www.asab.org/ethics). All procedures were strictly non‐invasive and excluded feeding, capture, experimentation or any physical contact. The research protocols were reviewed and approved by the relevant Thai authorities, including the Department of National Parks, Wildlife and Plant Conservation and the National Research Council of Thailand, which also granted permission for data collection and sample export under a benefit‐sharing agreement (permit numbers: 0002/4137, 0402/2798).

### 2.3 Urinary CTX‐I Analyses

The concentration of urinary CTX‐I was determined using a commercially available sandwich ELISA kit (Urine BETA CrossLaps [CTX‐I], REF AC‐05F1) provided by Immunodiagnostic Systems (IDS). This assay utilizes two highly specific antibodies for the detection of C‐terminal telopeptide fragments of Type I collagen in human urine, and its suitability in urine in bonobos has been previously validated (Behringer et al. 2024).

To evaluate potential urine matrix effects and optimal sample dilution in Assamese macaques, we performed serial dilutions on four randomly selected urine specimens in parallel with the ELISA standard curve, using CrossLaps Standard 0, in accordance with the manufacturer's protocol. Samples were tested undiluted and at dilution factors of 1:2, 1:4, and 1:8. The resulting dilution series exhibited parallelism with the standard curve, indicating negligible interference from urinary constituents (ESM Figure 1). Optimal assay performance was achieved at a 1:5 dilution for all samples.

**Figure 1:**
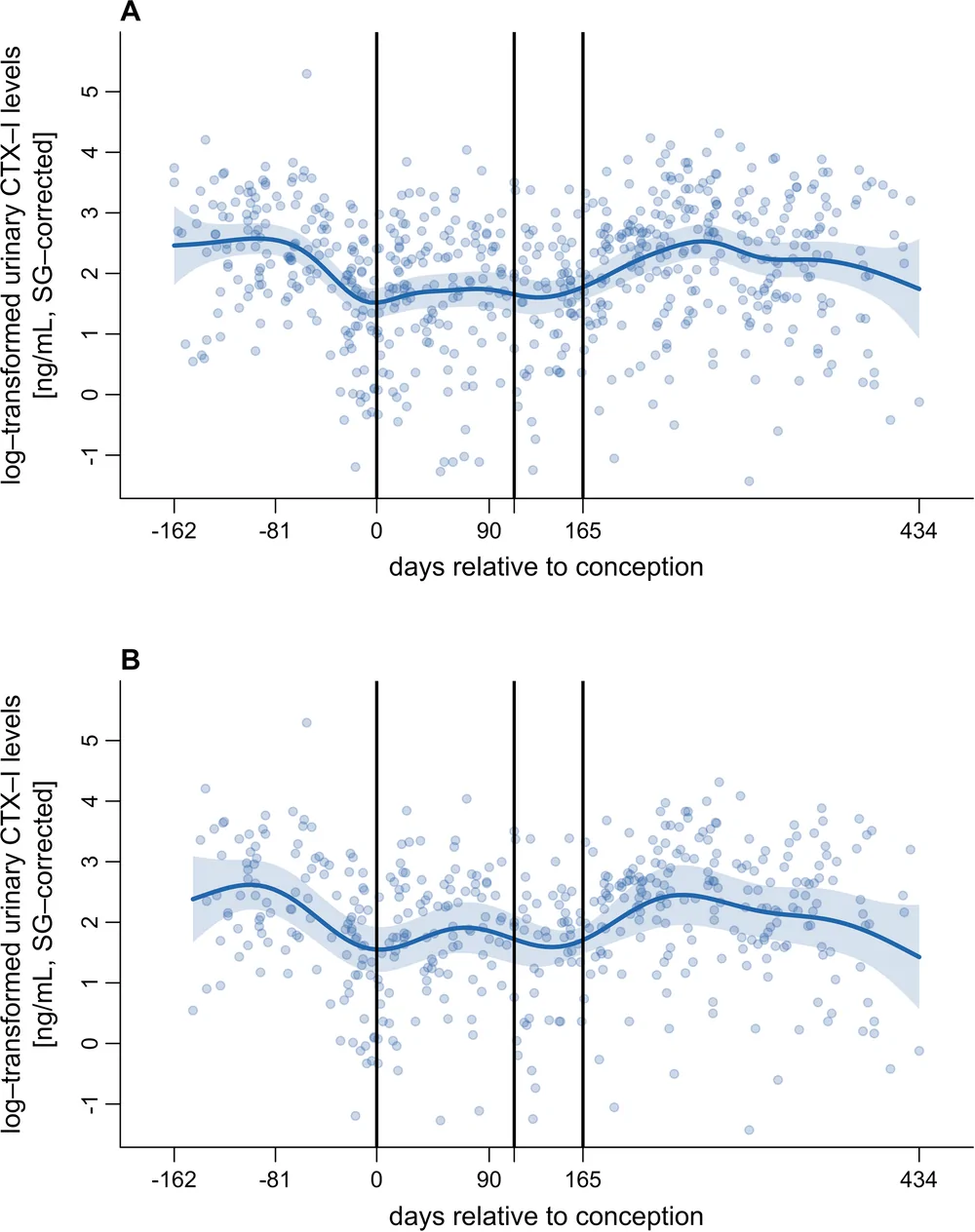
Non‐linear relationship between days relative to conception and urinary CTX‐I levels of 19 Assamese macaque females. (A) Includes all 19 females that gave birth in 2018, while (B) shows the subset of 13 females that gave birth in 2018 but did not have an offspring in 2017. The y‐axis displays log‐transformed urinary CTX‐I levels. The x‐axis represents days relative to the conception date (0), with negative values representing data prior to conception in 2018, when females were either nursing their 2017 offspring and/or cycling. Parturition (birth) occurs at day 165, after which females are nursing their 2018 offspring. Vertical solid lines denote reproductive landmarks: conception (0), onset of the final trimester (100), and parturition (165). The smooth term for days relative to conception in panel (A) indicates elevated CTX‐I levels both before conception (days −162 to −81) and during lactation (from day 165 onwards). However, results in (B) show that pre‐conception CTX‐I levels remain high and decrease in the 3 months towards conception even when females that gave birth in 2017 are excluded. [Color figure can be viewed at wileyonlinelibrary.com]

All samples were run in duplicates following IDS manual. Briefly, 10 μL of standards, controls, and diluted samples were dispensed into streptavidin‐coated microplate wells. Subsequently, 150 μL of a combined antibody solution, comprising biotinylated and peroxidase‐conjugated antibodies, was added. Plates were then sealed and incubated with continuous shaking (300 rpm) for 2 h at room temperature, protected from light. After incubation, wells were aspirated and rinsed five times using 300 μL of washing buffer. Next, 100 μL of chromogenic substrate was introduced, followed by a 15‐min incubation under the same conditions. The enzymatic reaction was terminated by addition of 100 μL sulfuric acid. Absorbance readings were acquired at 450 with 650 nm reference correction using a microplate reader.

Reported values were normalised to urine SG and expressed in nanograms per millilitre (ng/mL) corrected for SG.

### 2.4 Data Processing and Variable Definition

The dataset comprised 19 females who gave birth to an infant in 2018. Among these, two infants died at 208 and 269 days of age, respectively. Of the 19 females, six also had an offspring in the previous year (2017, Table 1), while the remaining thirteen had no offspring in 2017. Four of the six infants born in 2017 died at 27, 244, 245, and 353 days of age, respectively, whereas two survived. Sample collection took place between June 29, 2017 and January 17, 2019. Thus, the dataset captures at least a proportion of the lactation period for the 2017 infants, but does not encompass the pregnancies that resulted in the 2017 birth (2017 infants were born between March 31 and June 4, 2017). Data collection covers the entire gestation and lactation periods for the 2018 infants.

The estimated conception date for each pregnancy was determined by subtracting 165 days, which is the average gestation length in Assamese macaques (Fürtbauer et al. 2010; Shivani et al. 2025) from the recorded date of birth. For each sampling event, the number of days relative to this estimated conception date was calculated. Thus, the estimated conception date was assigned to 0 for all females. Days before this date had been assigned a negative direction in the timeline (with negative values). Conversely, days following the estimated conception date, corresponding to pregnancies with 2018 offspring or lactation to nurse these offspring, had positive values. Therefore, the variable ‘days relative to conception’ ranged from −162 to 434 days.

Due to mortality among offspring (four in 2017 and two in 2018), we created a binary variable ‘nursing status’. Nursing status was inferred based on offspring presence and survival at the time of sampling. Females were categorised as ‘nursing’ if they had a surviving offspring born in 2017 or in 2018 on the sampling date; otherwise, they were categorised as ‘not nursing’. Although the continuation of nursing into subsequent pregnancies in macaques remains a subject of debate (e.g., Dittus and Baker 2024), we assigned females during their pregnancies to the ‘not nursing’ category as suckling rates should at least be dramatically reduced as compared to the early phases of lactation (Berghänel et al. 2016). In other words, a female with a living infant and not being pregnant was categorised as nursing, and the day after the death of the offspring, the female was categorised as not nursing. While physiological adaptation such as downregulation of milk production may require time, as observed in humans in which these processes can span weeks post‐infant loss (Parish and Doherty 2021), species‐specific data for macaques are lacking. Consequently, the immediate reassignment to ‘non‐nursing’ status post‐infant death represents a conservative approach, minimising the risk of overestimating lactation effects on urinary CTX‐I levels, which may otherwise bias results in favour of our prediction. Although introducing a grace period might better approximate physiological adaptations, it would rely on arbitrary assumptions and risk inflating the ‘nursing’ sample size.

The time of sample collection was converted to minutes elapsed since midnight. Predictor variables, including time of sample collection, days relative to conception, and the number of offspring a female had prior to the 2018 infant, were mean‐centred and standardised to two standard deviations. This transformation procedure ensures that all predictors are expressed on a comparable scale, facilitating model convergence and interpretation (Araya‐Ajoy et al. 2015). Lastly, we applied a logarithmic transformation to the response variable urinary CTX‐I levels to achieve a more symmetrical distribution.

### 2.5 Statistical Analyses

All data preparations, analyses, and plotting of data were performed using R (R Development Core Team 2023) in RStudio (R Studio Team 2020).

Prior to fitting the generalised additive models (GAMs), we assessed multicollinearity among predictor variables by calculating the Variance Inflation Factor (VIF), using the vif() function from the car package (version 3.1.2; [Fox et al. 2019]). To test for potential collinearity between the predictors days relative to conception and day of the year (DOY), we first assessed VIF using a linear model including both predictors. All VIF values were low (max VIF = 1.82), indicating no problematic linear collinearity among predictors. However, concurvity diagnostics in the GAM including both smooths revealed substantial nonlinear overlap between the two time‐related terms (observed concurvity > 0.9), which is expected given the seasonal timing of conceptions. These results suggest that the smooth for days relative to conception shares a large proportion of its structure with other time‐related smooths—most likely because conceptions occur at similar times each year, aligning the reproductive and seasonal timescales. Therefore, we evaluated the effects of reproductive timing and seasonality in separate and combined models: (i) a reproduction‐only model with days relative to conception, (ii) a combined model including both days relative to conception and DOY, and (iii) a season‐only model with DOY. This approach allowed us to determine whether apparent reproductive effects could be explained by seasonal variation in CTX‐I levels.

We ran GAMs to investigate the non‐linear relationship between urinary CTX‐I levels and days relative to conception. First, we ran the model with the full dataset, including all females, irrespective of have given birth to an offspring in the previous season or not. Six females, who delivered an offspring in 2018, also had given birth to a surviving infant in 2017. In a follow‐up analysis, we re‐ran the GAM on a reduced dataset including only those 13 females who did not give birth in 2017 to exclude the noise produced by nursing the 2017 infants.

Models were fitted using the gam() function from the mgcv package (version 1.9.1, Wood 2011), with log‐transformed urinary CTX‐I levels as the response variable. We incorporated several predictor variables in the models: (i) a smooth term for days relative to conception, facilitating flexible modelling of its non‐linear relationship with urinary CTX‐I levels, (ii) time of day (min) to capture the circadian pattern of CTX‐I excretion previously documented in other species (Aoshima et al. 1998; Behringer et al. 2024; Tian and Ming 2022), and (iii) nursing status as a categorical predictor, based on the hypothesis that lactation is associated with higher urinary CTX‐I levels compared to other reproductive states (Liesegang et al. 2000; Wysolmerski 2002). All continuous variables were entered into the models mean‐centred and standardised to two standard deviations (see Araya‐Ajoy et al. 2015).

To account for the repeated measures design and for individual variability in baseline urinary CTX‐I levels, a random intercept for individual females was included. Whether the inclusion of a random slope for days relative to conception across females would improve model fit was tested by comparing models with and without this term, using the compareML() function in the itsadug package (version 2.4.1, van Rij et al. 2022), employing Akaike Information Criterion (AIC) for model selection. We applied restricted maximum likelihood (REML) for smoothness estimation, allowing flexible nonlinear fits while accommodating individual‐level variability.

To identify the appropriate degree of flexibility for the smooth term representing days relative to conception, we conducted a grid search combined with cross‐validation. Candidate models were fitted across a range of basis dimensions (k) from 5 to 50. For each k, the dataset was randomly split into training (80%) and validation (20%) subsets. Models were fitted on the training data using maximum likelihood (ML) estimation to facilitate comparability, and predictive accuracy was assessed on the validation set using mean squared prediction error (MSPE). The optimal k was chosen as the value minimising the MSPE, and this parameter was selected for the final model.

To verify that the assumptions of the GAM models were met, we performed a series of diagnostic checks. Visual inspection of the smooth terms was conducted using the draw function of the gratia package (version 0.9.2, Simpson 2024) to confirm the modelled relationships were plausible and adequately captured non‐linear trends. Residual diagnostics were carried out using the appraise() function from the same package (version 0.9.2, Simpson 2024) to evaluate residuals homoscedasticity, independence, and detect potential outliers. Additionally, we applied the gam.check() function from the mgcv package (version 1.9.1, Wood 2011) to evaluate model convergence, the adequacy of the basis dimension (k), and the distribution of residuals. Unless stated otherwise, all model assumptions were met.

Due to the results the previously described models revealed, we fitted several post‐hoc models: To investigate whether the observed non‐linear variation in urinary CTX‐I levels was driven by seasonal effects rather than days relative to conception alone, we fitted additional GAMs including smooth terms for both days relative to conception and DOY as predictors. The variable DOY (1–365) was derived from the date of sample collection, representing annual cyclical variation. We applied cross‐validation to identify the optimal number of basis functions (k) for the smooth terms of days relative to conception and DOY, respectively. In the first model, we included smooth terms for both days relative to conception and DOY (with a cyclic cubic regression spline), along with time of day, nursing status, a random intercept for individual identity, and random smooths for the interaction between each smooth term and ID to capture individual variation. We additionally tested for an interaction between days relative to conception and DOY using a tensor product smooth.

To isolate the effect of seasonality, we also fitted a GAM including only DOY (with optimal k determined by cross‐validation), time of day, and random effects (random intercepts and random smooths for DOY by individual ID). Finally, we assessed whether seasonal changes in urine concentration, rather than seasonal patterns in urinary CTX‐I levels, could explain variation in CTX‐I levels. We modelled specific gravity (SG; log‐transformed), which reflects the water content of urine and thus its concentration, as a function of DOY and time of day, including random effects. The predicted seasonal variation in SG (i.e., the fitted values from this model) was then incorporated as a covariate into the CTX‐I model to evaluate whether adjusting for SG reduced the seasonal effect on urinary CTX‐I levels. Together, these post hoc analyses enabled us to disentangle reproductive from seasonal drivers of CTX‐I variation and to evaluate potential confounds by seasonal fluctuations in urine concentration.

## 3 Results

We fitted a GAM model including a smooth term for days relative to conception (k = 37), time of day, nursing status, a random intercept for individual females and a random slope for days relative to conception. Comparison of this model with the corresponding null model (including time of day and the random effects) showed that inclusion of predictor variables improved the model fit (*χ* ^2^ = 20.32, df = 1, *p* < 0.001; delta AIC = 48.10). Including random slopes for days relative to conception slightly improved model fit as compared to a model with a random intercept for individual identity (delta AIC = 5.62), but this difference was not statistically significant (*χ* ^2^ = 2.19, df = 2, *p* = 0.111). To preserve model parsimony, the simpler random intercept model was retained for all subsequent analyses. The random intercept model was significantly better than the same model without the random intercept (*χ* ^2^ = 13.28, df = 1, *p* < 0.001; delta AIC = −38.72), suggesting substantial individual differences in their average urinary CTX‐I excretion.

The random intercept GAM (Table 2) explained 24.9% of the deviance in log‐transformed urinary CTX‐I levels (adjusted *R* ^2^ = 0.22, scale estimate = 0.916). Urinary CTX‐I levels had a negative association with time of day (*β* = −0.423, SE = 0.076, *p* < 0.001), indicating a decrease in urinary CTX‐I levels towards nightfall. Nursing status was not a significant predictor (*β* = 0.017, SE = 0.159, *p* = 0.913).

**Table 2:** Results of the generalised additive random intercept models predicting log‐transformed urinary CTX‐I levels in female Assamese macaques.

	*β* (estimate)	SE (standard error)	*t* value	*p* value
Intercept	2.042	0.109	18.736	< 0.001
	2.008	0.175	11.506	< 0.001
Time of day	−0.423	0.076	−5.596	< 0.001
	−0.455	0.091	−4.986	< 0.001
Nursing state (reference: nursing)	0.017	0.159	0.109	0.913
	0.099	0.351	0.284	0.777

	Edf (estimated degrees of freedom)	Ref df (reference degrees of freedom)	*F*	*p* value
Smooth term days relative to conception	9.713	11.95	5.721	< 0.001
	6.952	7.683	5.529	< 0.001
Random intercept	13.174	18.0	2.918	< 0.001
	8.218	12.0	2.308	< 0.001

*Note:* Each cell of the table contains results from two models: the upper value refers to the full dataset (N = 19 females), and the lower value refers to the reduced dataset (N = 13 females) excluding individuals who had an offspring in 2017 (i.e., who may have been lactating prior to the 2018 pregnancy). The reduced model tests whether observed pre‐conception CTX‐I patterns are attributable to prior lactation. All models included the same fixed and random effects structure.

The smooth term for days relative to conception predicted urinary CTX‐I levels (edf = 9.713, *F* = 5.72, *p* < 0.001), suggesting a nonlinear relationship between days relative to conception and urinary CTX‐I levels. The high estimated degrees of freedom (edf) indicated that this term captured considerable nonlinearity in the data, reflecting distinct physiological changes at different stages of the reproductive year (Figure 1A). Basis dimension diagnostics confirmed that the selected basis dimension (k = 20) was appropriate to represent complexity of the data. Additionally, the random intercept for individual females was significant (edf = 13.174, *F* = 2.918, *p* < 0.001), indicating substantial individual variability in urinary CTX‐I levels.

To exclude the possibility that elevated urinary CTX‐I levels prior to conception in 2018 were driven by lactating females with offspring born in 2017, we replicated the GAM analysis on a reduced dataset including only the 13 females who had not given birth in 2017. Cross‐validation identified 9 as the optimal basis dimension (k) for the smooth term of days relative to conception. Using k = 9, we fitted a GAM with the same structure as the main model, including a smooth term for days relative to conception, the same linear predictors (time of day, nursing status), and a random intercept for individual females (Table 2). The non‐linear association between days relative to conception and urinary CTX‐I levels remained robust in this subset (Figure 1B), with a similar shape to that observed in the full dataset: about two to 3 months prior to conception urinary CTX‐I levels started to decrease from maximal levels to stay relatively low throughout pregnancy. Across pregnancy, highest levels were estimated prior to the last trimester. After parturition, urinary CTX‐I levels increased steadily to reach maximal levels, comparable to those observed prior to conception. These results (which are similar using the full and the reduced datasets) suggest that the elevated CTX‐I levels prior to conception were not solely attributable to lactation of a few females, but likely reflected other physiological or seasonal processes that affect urinary CTX‐I levels.

In the post hoc additive model including smooths for both days relative to conception (k = 42) and DOY (k = 22), only the smooth term for DOY predicted urinary CTX‐I levels (Table 3), while the effect of days relative to conception did not. The model explained 32.8% of deviance (adjusted *R* ^2^ = 0.27). Including an interaction between days relative to conception and DOY did not significantly improve model fit (delta AIC = 2.84, *χ* ^2^ = 0.57, df = 2, *p* = 0.567).

**Table 3:** Results of the generalised additive random intercept model containing smooths for days relative to conception and day of the year (DOY) as predictors for log‐transformed urinary CTX‐I levels in female Assamese macaques.

	*β* (estimate)	SE (standard error)	*t* value	*p* value
Intercept	2.050	0.110	18.622	< 0.001
Time of day	−0.434	0.074	−5.848	< 0.001
Nursing state (reference: nursing)	−0.003	0.162	−0.019	0.985

	Edf (estimated degrees of freedom)	Ref df (reference degrees of freedom)	*F*	*p* value
Smooth term days relative to conception	7.326	9.286	1.450	0.156
Smooth term DOY	4.549	20.00	0.538	< 0.001
Random intercept	13.372	18.00	3.133	< 0.001
Slope for days relative to conception	2.534	72.00	0.041	< 0.001
Slope for DOY	19.407	57.00	0.605	< 0.001

*Note:* The model was fit on the full dataset (*N* = 19 females).

A season‐only model including the smooth for DOY (k = 47), time of day, and random effects explained 30.1% of the deviance (adjusted *R* ^2^ = 0.25; for details see ESM). The seasonal pattern of urinary CTX‐I levels exhibited two peaks annually, a minor one around February and a major one around August and September (Figure 2). Modelling SG revealed significant seasonal variation in urine concentration (edf = 12.83, *F* = 2.52, *p* < 0.001; for details ESM Supporting Information S1: Figure 2), but including this predicted SG component as a covariate did not substantially affect the seasonal smooth of urinary CTX‐I (residual SG: *β* = −7.53, SE = 16.07, *p* = 0.639; ESM Supporting Information S1: Figure 2), indicating that seasonal variation in urinary CTX‐I levels is not driven by changes in urine concentration.

**Figure 2:**
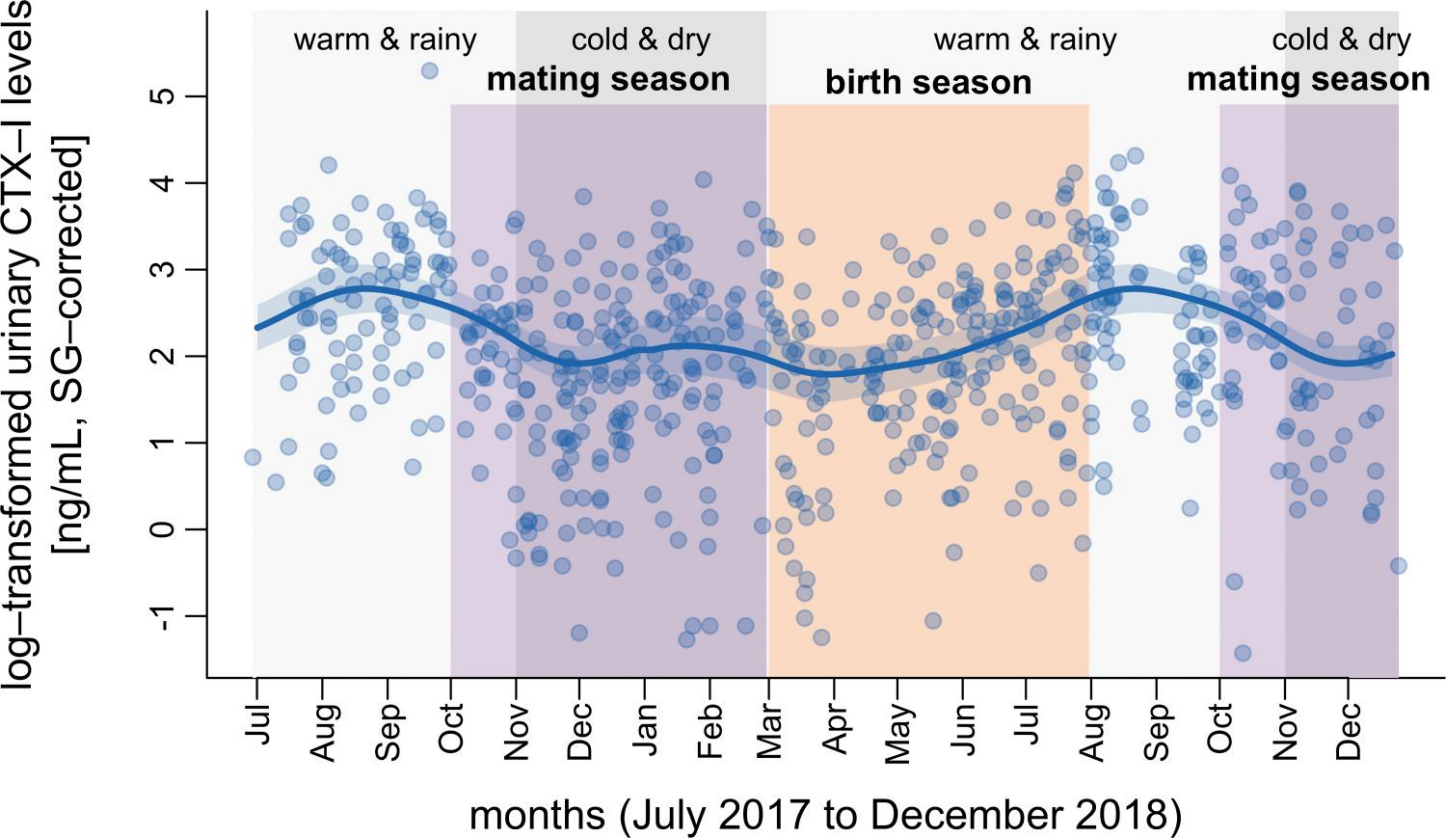
Seasonal variation in log‐transformed urinary CTX‐I levels in female Assamese macaques from July 2017 to December 2018. Dots represent raw data, with each point corresponding to an individual urine sample. The solid blue line shows the predicted seasonal trajectory of urinary CTX‐I levels derived from a generalised additive model including day of year (DOY) as a smooth term, controlling for specific gravity (SG), time of day, and individual identity. The shaded area indicates the 95% confidence interval around the smooth term. The x‐axis displays months across the 2‐year observation period. Coloured blocks indicate the mating and birthing seasons (Touitou et al. 2021a), while grey shaded areas depict wet and dry seasons (Richter et al. 2016). [Color figure can be viewed at wileyonlinelibrary.com]

## 4 Discussion

Viewed from the onset of a reproductive event at conception, urinary CTX‐I levels of female Assamese macaques remained relatively low during pregnancy with a minor peak roughly in the middle of the period to then increase sharply after parturition and reaching an absolute maximum 3 months into lactation. The other peak in urinary CTX‐I levels 3 months prior to conception could not be attributed to lactation with the previous offspring, because it remained after excluding those females that had given birth the previous year. Thus, the observed variation in bone resorption is not solely attributable to lactation. Our post‐hoc analyses suggest that instead of being mainly driven by changing calcium demands of reproductive life‐history phases, the urinary CTX‐I pattern may reflect a seasonal physiological rhythm with elevated levels in August, September, and October, possibly linked to broader reproductive endocrine changes or ecological drivers.

### 4.1 Endocrine Seasonality and Bone Resorption

As seasonal breeders, Assamese macaques experience fairly predictable annual fluctuations in reproductive and metabolic hormones such as estrogen, progesterone, triiodothyronine, and cortisol (Behringer et al. 2025; Fürtbauer et al. 2010; Touitou et al. 2021a, 2021b), all of which influence bone metabolism (Hart et al. 2020; Kini and Nandeesh 2012; Mallorie and Shine 2022). For example, these females do not have ovarian cycles prior to the mating season in October (Fürtbauer et al. 2010), leading to overall low estrogen and progesterone levels across individuals regardless of lactation status. Estrogens inhibit bone resorption (Hart et al. 2020), and low estrogen levels remove this inhibitory effect, leading to increased osteoclast activity and an imbalance in bone remodelling that favours resorption over formation (Kini and Nandeesh 2012; Mallorie and Shine 2022). Progesterone also contributes to bone health by stimulating osteoblast activity directly, via its receptors on osteoblasts, and indirectly, by competing with glucocorticoids for osteoblastic receptor binding (Kini and Nandeesh 2012). Consequently, when progesterone levels are low, bone formation is reduced.

The combination of sustained low estrogen and progesterone environment prior to the mating season may contribute to both enhanced osteoclast activity and reduced osteoblast function, thereby potentially promoting increased bone resorption and urinary CTX‐I levels. However, the similarity in CTX‐I levels between pre‐conceptive and lactating females suggests that seasonal endocrine patterns may interact with, or partially mask, reproductive‐state‐specific effects on bone metabolism.

While CTX‐I primarily reflects degradation of type I collagen associated with osteoclast‐mediated bone resorption (Herrmann and Seibel 2008), lactation is additionally characterised by osteocytic perilacunar remodelling (Tsourdi et al. 2018). Because CTX‐I reflects systemic collagen degradation products rather than cell‐specific activity, the relative contribution of osteocyte‐driven matrix remodelling to circulating CTX‐I concentrations has not been directly quantified. Thus, CTX‐I is interpreted here as an integrated marker of skeletal resorption, capturing overall bone mobilisation during lactation.

Metabolic hormones such as triiodothyronine (T3) and cortisol can also influence bone health. For example, increased T3 levels stimulate both bone formation and resorption by increasing osteoblast and osteoclast activity, but can ultimately reduce bone density by accelerating bone turnover (Hart et al. 2020; Mallorie and Shine 2022). Cortisol generally promotes bone resorption and inhibits bone formation through multiple mechanisms (Hart et al. 2020; Mallorie and Shine 2022). In the same female Assamese macaques in 2018, both T3 and cortisol levels were found to decrease steeply from late gestation to early lactation (Touitou et al. 2021b), which should be associated with decreased CTX‐I levels after parturition and therefore suggests that fluctuations in these metabolic hormones are unlikely to drive the observed seasonal increase in CTX‐I levels in our study.

Altogether, our findings indicate that reproductive status alone does not explain variation in urinary CTX‐I levels across the reproductive cycle. Instead, these patterns likely reflect broader endocrine fluctuations, although not all hormones contribute equally to these fluctuations. Future studies should focus on identifying which specific hormonal changes drive this seasonal physiology.

### 4.2 Ecological Influences on Bone Resorption

Environmental cues influencing seasonal physiology could further modulate urinary CTX‐I patterns. For example, vitamin D synthesis in the skin depends on exposure to UV‐B radiation from sunlight (Bogh et al. 2011). UV‐B converts 7‐dehydrocholesterol in the skin to vitamin D_3_, which is subsequently metabolised in the liver to 25‐hydroxyvitamin D_3_ and in the kidneys to its active form, 1,25‐dihydroxyvitamin D_3_. This active metabolite enhances intestinal calcium absorption and thereby supports bone health (Holick 2011). In other words, when vitamin D levels are insufficient, calcium absorption is reduced, which can cause compensatory bone resorption to maintain serum calcium levels, processes reflected in elevated urinary CTX‐I levels.

UV‐B availability varies by geographic location, season, and weather conditions (Holick 2014). For macaques living in the tropics, vitamin D insufficiency does not so much result from reduced solar irradiance in winter, but may additionally be influenced by changes in cloud cover that reduces UV‐B exposure. Vitamin D insufficiency is common in humans across different regions in Thailand (Chailurkit et al. 2011), which suggests potential ecological constraints on wildlife as well.

Vitamin D can be stored in fat and muscle tissue in its 25‐hydroxyvitamin D (25(OH)D) form (Carrelli et al. 2017; Lawson et al. 1986). However, this storage is not readily mobilised and may not serve as an accessible reservoir (Rybchyn et al. 2020; Ziaie et al. 2016). Alternatively, circulating 25(OH)D has a notably long half‐life, ranging from 15 to 50 days and extending up to 89 days in some individuals (Clements et al. 1987; Datta et al. 2017; Rybchyn et al. 2020). This feature provides a temporal buffer that may help maintain vitamin D status during periods of low UV‐B exposure. Nonetheless, these circulating stores are finite and can be depleted during extended periods of limited sunlight, such as the long wet season in North‐Eastern Thailand (April–October; Grassman et al. 2005). Consistent with this, our data revealed that Assamese macaques exhibit markedly high urinary CTX‐I levels, reflecting high bone resorption, towards the end of the rainy season, particularly in September and October. One possible explanation is that diminishing vitamin D availability over the course of the rainy season may contribute to increased skeletal catabolism, potentially restricting opportunities for bone recovery and mineralisation during this period.

As a plausible response to reduced UV‐B exposure, some primates have been reported to become more arboreal in the wet season when cloud cover prevents UV‐B exposure (*Pan troglodytes*, Takemoto 2004; *Trachypithecus leucocephalus*, Zheng et al. 2021). Assamese macaques at the study site do not adopt this strategy. They do not become more arboreal since they spend only a small fraction of their time on the ground year‐round (Schülke et al. 2011), but they also do not get closer to the light as they do not increase their use of the canopy layer above 12% of their activity time in the wet months from April through October. Actual sunbathing has been observed in December and January only after the coldest nights of the year and is unlikely to mainly serve the enhanced UV‐B exposure.

In summary, seasonal variation in cloud cover and thus UV‐B exposure may lead to reduced vitamin D synthesis and, over time, lower vitamin D availability. This, in turn, may limit calcium absorption and increase bone resorption, thereby potentially contributing to the observed seasonal changes in urinary CTX‐I levels in Assamese macaques. Future studies should more directly investigate how ecological constraints shape vitamin D status and bone metabolism in wild primates.

### 4.3 Time of Day Affects CTX‐I Levels

Our findings revealed a diurnal decline in urinary CTX‐I levels, with concentrations decreasing over the course of the day. This pattern confirms the presence of a circadian rhythm in bone resorption in Assamese macaques, serving as a biological validation because it mirrors observations in humans and other primates (Aoshima et al. 1998; Behringer et al. 2024; Tian and Ming 2022). These consistent patterns across species suggest a conserved circadian regulation of bone turnover. One proposed mechanism involves the influence of cortisol. While seasonal variation in cortisol levels is unlikely to explain the observed annual CTX‐I pattern (see above), its well‐established diurnal rhythm may still contribute to short‐term (within‐day) fluctuations in bone remodelling activity (Mallorie and Shine 2022). Our results underline the importance of accounting for time of day when collecting urine samples and interpreting urinary CTX‐I measurements.

### 4.4 Interindividual Variability in CTX‐I Levels

We observed significant individual differences in urinary CTX‐I levels, independent of reproductive state, as reflected by the improved model fit when including a random intercept for individual identity. While this indicates variability among individuals, it does not alone demonstrate stable traits or repeatability over time and contexts. Nevertheless, these differences could arise from various intrinsic or extrinsic factors not accounted for in our dataset. Possible influences on individual bone resorption include genetic predispositions, age‐related changes, long‐term energetic conditions, the type of food that animals consume, or environmental variables such as social dynamics and ecological factors (Jayo et al. 1994; Papageorgiou et al. 2017; Riggs et al. 2002; Seeman 2001). Additionally, consistent behavioural patterns, including differences in substrate use or locomotor pattern, might affect mechanical loading on the skeleton and thereby modulate bone turnover (Ruff and Larsen 2014; Schriefer et al. 2005). Future studies should explore whether these individual differences are stable over time and which specific factors underlie this variation, as they may reveal determinants of skeletal health in wild primates.

In summary, our study shows that urinary CTX‐I levels in wild female Assamese macaques vary across the year in a pattern shaped more by season than by pregnancy and lactation. Longitudinal sampling revealed peaks in urinary CTX‐I levels with seasonal timing of these peaks suggesting an influence of environmental or endocrine cycles rather than solely reproductive calcium demands. By demonstrating that urinary CTX‐I levels can reliably be measured non‐invasively in wild primates and used to monitor bone resorption over time, our results provide a framework for future studies examining how bone metabolism is modulated by ecological and physiological factors across the annual cycle.

## Author Contribution

**Verena Behringer:** conceptualisation (equal), investigation (equal), methodology (equal), validation (lead), writing − original draft (lead), writing − review and editing (equal). **Suchinda Malaivijitnond:** project administration (equal), writing − review and editing (equal). **Suthirote Meesawat:** project administration (equal), writing − review and editing (equal). **Ruth Sonnweber:** formal analysis (lead), visualisation (lead), writing − review and editing (equal). **Tracy L. Kivell:** Conceptualisation (equal), funding acquisition (equal), validation (equal), writing − review and editing (equal). **Oliver Schülke:** conceptualisation (equal), funding acquisition (equal), methodology (equal), project administration (equal), resources (equal), writing − review and editing (equal). **Julia Ostner:** conceptualisation (equal), funding acquisition (equal), investigation (equal), methodology (equal), project administration (equal), resources (equal), writing − review and editing (equal).

## Conflicts of Interest

The authors declare no conflicts of interest.

## Supporting information


Supporting File

## Data Availability

The data that support the findings of this study are available from the corresponding author upon reasonable request.
