# Supplementary material for: A Year in the Bones: Investigating Bone Resorption Across a Reproductive Year in Wild Female Assamese Macaques
Source: J Anim Physiol Anim Nutr (Berl). 2026 Aug 31;110(6):1006–19. doi: 10.1111/jpn.70108 (PMC13768894; doi:10.1111/jpn.70108)
Supplement: Supplementary file 1 [file JPN-110-1006-s001.pdf]

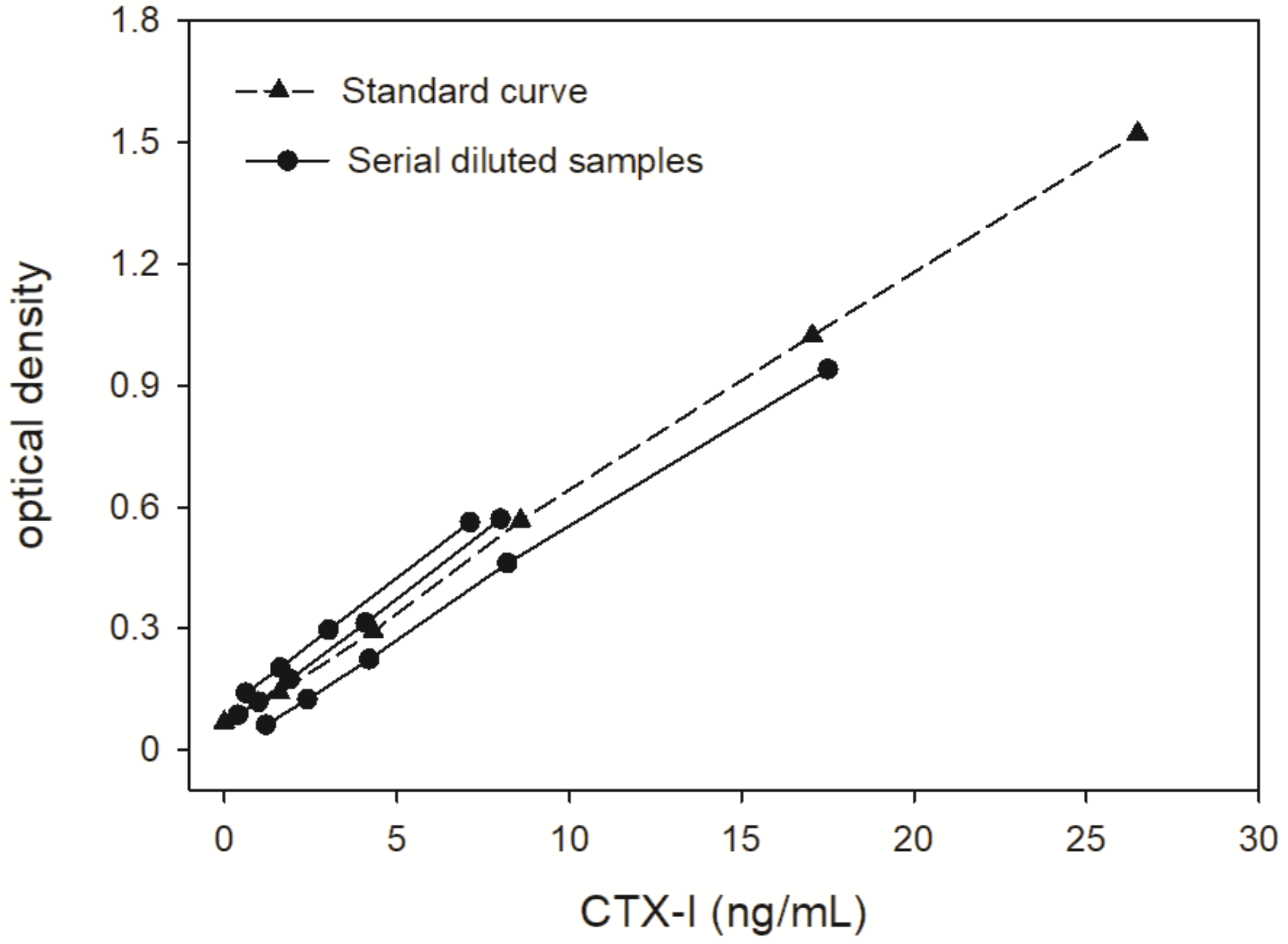

Figure S1: Concentrations of standards and serial dilutions of samples (ng/mL). Linearity of four urine Assamese macaque urine samples. Standard curves (dotted lines, filled triangles) and serial diluted urine samples (straight lines, filled circles).

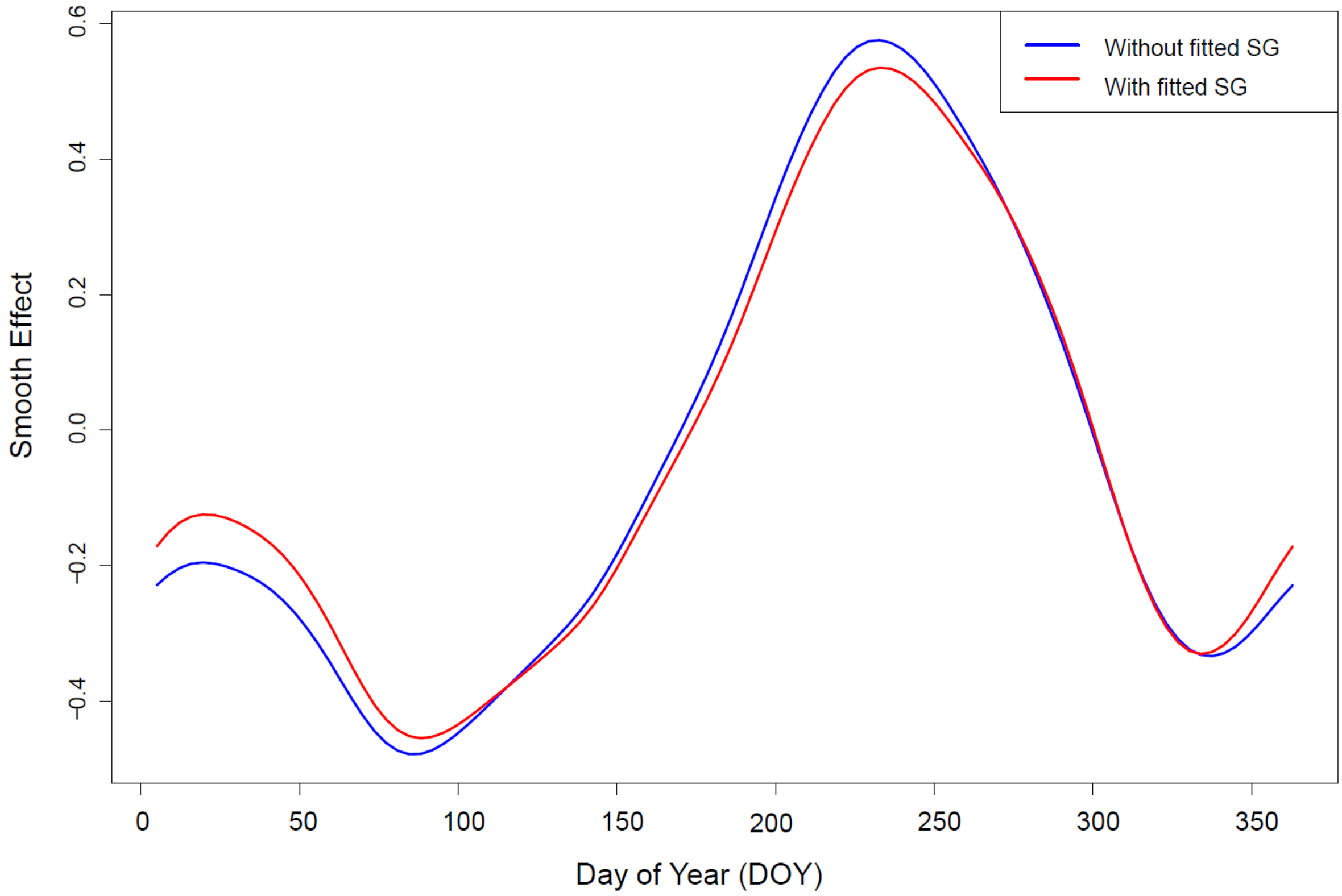

Figure S2. Seasonal variation in urinary specific gravity (SG) of female Assamese macaques. The GAM model revealed significant seasonal variation in urine concentration (edf = 12.83, $F = 2.52$, $p < 0.001$). Including the predicted SG component as a covariate in the CTX-I model did not substantially affect the seasonal smooth of urinary CTX-I levels (residual SG: $\beta = -7.53$, SE = 16.07, $p = 0.639$), indicating that seasonal variation in urinary CTX-I is not driven by changes in urine concentration.
